# The Microvascular Gap Junction Channel: A Route to Deliver MicroRNAs for Neurological Disease Treatment

**DOI:** 10.3389/fnmol.2017.00246

**Published:** 2017-08-04

**Authors:** Dominique Thuringer, Eric Solary, Carmen Garrido

**Affiliations:** ^1^INSERM U1231, Université de Bourgogne Franche Comté Dijon, France; ^2^INSERM U1170, Institut Gustave Roussy Villejuif, France

**Keywords:** targeted therapy, microRNA, gap junction, blood capillary, connexin43, neurological disorders, glioblastoma

## Abstract

Brain microvascular endothelial cells (BMECs) separate the peripheral blood from the brain. These cells, which are surrounded by basal lamina, pericytes and glial cells, are highly interconnected through tight and gap junctions. Their permeability properties restrict the transfer of potentially useful therapeutic agents. In such a hermetic system, the gap junctional exchange of small molecules between cerebral endothelial and non-endothelial cells is crucial for maintaining tissue homeostasis. MicroRNA were shown to cross gap junction channels, thereby modulating gene expression and function of the recipient cell. It was also shown that, when altered, BMEC could be regenerated by endothelial cells derived from pluripotent stem cells. Here, we discuss the transfer of microRNA through gap junctions between BMEC, the regeneration of BMEC from induced pluripotent stem cells that could be engineered to express specific microRNA, and how such an innovative approach could benefit to the treatment of glioblastoma and other neurological diseases.

## Introduction

Human brain microvascular endothelial cells (BMECs) interact with astrocytes and pericytes to form a functional “neurovascular unit” called the blood–brain barrier (BBB), which protects the central nervous system by preventing the transfer of circulating molecules from the bloodstream to the brain parenchyma. A drawback of this efficient barricade is its ability to restrict the transfer of potentially useful neurotherapeutic agents. In the recent years, microvascular endothelial cells derived from induced pluripotent stem cells (iPSC) were used to further explore BBB development and maintenance by co-culture with neural cells. These iPSC appeared also as a biological tools to screen neuropharmaceuticals (Lippmann et al., 2013, 2014a; Cecchelli et al., 2014; Minami et al., 2015; Katt et al., 2016; Appelt-Menzel et al., 2017; Yamamizu et al., 2017). Meanwhile, analysis of gap junction channels between heterotypic cells suggested promising applications by blocking or promoting microRNA transfer and delivery (Valiunas et al., 2005; Lemcke et al., 2015). This short review focuses on how we could deal with these specific aspects of the BBB biology to transfer therapeutic microRNAs to the brain.

## BMEC As A Gateway for Drug Delivery to the Brain

As compared to other endothelial cells, the highly polarized BMEC demonstrate specific features. They form circumferential tight junction complexes, establishing a high-resistance paracellular barrier to small hydrophilic molecules and ions. The rate of transcytosis is low, although this remains the preferred pathway for the selective transport of plasma macromolecules. The expression of selective influx/efflux transporters such as ATP binding cassette (ABC) efflux transporters against concentration gradients is another characteristic aspect of these cells. Finally, the absence of leukocyte adhesion molecules, together with tight junctions, prevents the entry of peripheral immune cells in the absence of trauma or disease (Abbott et al., 2006; Chow and Gu, 2015). These BMEC specific features are a physical challenge and rate-limiting step for therapeutically targeting brain cells (Joshi et al., 2017). Importantly, the microvascular network of the brain is dense and so intricate that every neuron or glial cell is less than 20 μm from a blood capillary. In other words, a molecule that would cross the BBB will be immediately delivered to every neuron within the brain (Pardridge, 2002). The protective function of the BBB can be severely impaired during neurodegenerative and neuroinflammatory disorders, ischemic stroke and central nervous system (CNS) tumor development. An altered BBB may influence the treatment efficacy of these diseases with drugs that may not traverse the BBB to reach their target in the diseased brain while the delivery of others is hampered by disturbed transport mechanisms (Schenk and de Vries, 2016; Reinhold and Rittner, 2017). In brain tumors, vasogenic edema, elevated intracranial pressure, hypoxia, and neo-angiogenesis also contribute to create a chaotic situation that affects drug bioavailability (Saunus et al., 2017).

Small, lipophilic compounds and some hydrophobic molecules can cross the BBB. In contrast, biologic drugs such as nucleic acids, recombinant proteins, antibodies, and peptides are usually too large to be transferred through the BBB. This delivery could be promoted by re-engineering these large molecules into brain-penetrating neuropharmaceuticals (Pardridge, 2015a,b). Their delivery to the brain after intravenous injection could be also slightly improved by co-administration of low doses of a hyperosmolar solution (Gray et al., 2010; Kwon et al., 2010) or by disrupting the BBB with microbubble-enhanced ultrasound (Tan et al., 2016). Gene vectors have been injected directly into the brain to circumvent the BBB (Do Thi et al., 2004; Yang et al., 2013) but the innate difficulty of the method and the risks induced by such an approach make it hardly applicable over long-term clinical trials (Joshi et al., 2017).

Another strategy would be to control the functions of BMEC into the intact endothelium. These cells have a short time life when compared to other cerebral cell types (i.e., of about 2 months) and this life time could be further reduced by inflammation. The renewal of BMEC is ensured within few hours, either by cell division of neighboring endothelial cells or by the cell differentiation of circulating blood cells. Embryonic stem cells can differentiate into any cell type including endothelial cells (Levenberg et al., 2002; Wang et al., 2007; Kane et al., 2010; Nourse et al., 2010) and their properties are recapitulated by iPSC, which hold great promise for regenerative medicine (Kane et al., 2011; Kimbrel and Lanza, 2016). Such iPSC-derived endothelial cells have been combined with cardiomyocytes, and smooth muscle cells to improve cardiac function after acute myocardial infarction in a porcine model (Ye et al., 2014). It remains to be determined if they could modify the BBB composition, which would allow to engineer these cells, e.g., to express a specific microRNA or small silencing RNA (siRNA), before using them to repair an altered BBB while introducing new functions that facilitate drug delivery to the brain.

## Gap Junctional Intercellular Communications

The renewal of microvascular endothelial cells is followed by the rapid re-establishment of intercellular junctions. The gap junctional intercellular communication (GJIC) can only be established if the cells are closely joined by tight junctions [and especially express adhesion proteins such as zonulae occludens ZO-1 (Zhang et al., 2003; Sin et al., 2012; Lippmann et al., 2014b; Minami et al., 2015)]. For instance, adhesion of cells to an endothelial monolayer is usually achieved in less than 1 h, cell–cell communication is established in 1–2 h and the gap junctional shuttling of microRNA observed within 3 h *in vitro* (Thuringer et al., 2015b, 2016a). The gap junction proteins, namely connexins (Cx) Cx37, Cx40, and Cx43 are expressed in BMEC (Vis et al., 1998; De Bock et al., 2011, 2017; Kaneko et al., 2015; Bader et al., 2017) as well as in iPSC which display intercellular dye transfer as expected for GJIC (see below). Interestingly, the glioblastoma microenvironment up-regulates the gene expression of tight junction proteins in iPSC-derived BMEC (Minami et al., 2015). Healthy and diseased glial cells separated by 3–4 nm also form functional gap junctions (Baker et al., 2014; Stamatovic et al., 2016), mostly composed of Cx30 and Cx43 (Wallraff et al., 2006; De Bock et al., 2017). In all cases, remodeling of gap junctions occurs constantly with a high turnover rate; i.e., Cx typically have short half-life of about 1.5–6 h in mammalian cells (Laird, 2006; Herve et al., 2007). Heterocellular gap junctions formed by Cx43 are largely described between BMEC and glial cells *in vitro*, and induce barrier properties in non-brain blood vessels in transplantation studies (Janzer and Raff, 1987; Abbott et al., 2006). *In vivo*, the basal lamina may limit the formation of gap junctions in most of the brain vasculature but pathological situations such as glioblastoma cell invasion that degrade the basal lamina, increase the probability of developing such communications (Bart et al., 2000; Vajkoczy and Menger, 2004; Alves et al., 2011).

The GJIC established between cancer and healthy cells permits the direct transfer of cytosolic messengers, including single strand of 22-nucleotide non-coding RNA that modify gene expression and functions of recipient cells (Valiunas et al., 2005; Lemcke et al., 2015). The GJIC-mediated intercellular transfer of mature microRNA was reported to be Cx-dependent (Zong et al., 2016). The permeability of Cx-formed gap junctions to microRNAs, responds to the following order: Cx43 > Cx26/30 > Cx26 > Cx31 > Cx30 = Cx-null with Cx43 having high permeability and Cx30 being poorly permeable to microRNAs (Zong et al., 2016). This is consistent with previous reports that Cx30 channels are impermeable to negatively charged molecules; i.e., all nucleotides including microRNA being anionic at physiological pH. We have detected *in vitro* microRNA exchanges between human microvascular endothelial (HMEC) and colon cancer cells through Cx43-formed gap junctions (Thuringer et al., 2016b). More specifically, the transfer of miR-145 from HMECs to tumor cells was observed to inhibit angiogenesis and tumor growth. Similar exchanges were observed between HMECs and glioblastoma cells, highlighting the crucial role of Cx43-formed gap junctions in the regulation of BBB genes (Thuringer et al., 2016a).

The opening of gap junction channels can also lead to the transfer of pathologic microRNAs such as miR-5096 that promotes glioblastoma cell invasion (Hong et al., 2015; Thuringer et al., 2016a, 2017). We evidenced such a transfer between HMEC and glioblastoma cell lines, indicating that microRNAs have to be carefully selected and evaluated for their ability to favor tumor regression. Of note, miR-5096 was shown to down-regulate Cx43 expression in glioma cells. More specifically, in co-culture experiments, membrane GJIC plaques disappeared in glioma cells while they drastically increased in HMEC (Thuringer et al., 2016a), maybe explaining why BBB remains hermetic to glioma metastasis (Blecharz et al., 2015; Steeg, 2016; Saunus et al., 2017). Despite of the absence of GJIC, glioma cells were still able to transfer miR-5096 to HMEC through the release of exosomes (Thuringer et al., 2017). Whether other microRNAs could be transferred from glioma cells to BMEC or reciprocally and how it could interfere with invasion process remains to be determined. Additionally to forming GJIC, hemichannels also participate in cell–cell communication (De Bock et al., 2017): firstly, by behaving as docking sites for exosomes, allowing therefore direct transfer of exosomal microRNA to neighboring cells (Soares et al., 2015); and secondly, by secreting microRNA into the intercellular spaces, compensating therefore the loss of glial endfeet of proliferating cancer cells and the increase in the perivascular space observed in tumor satellites (Noell et al., 2012).

Thus, the GJIC could be used to transfer therapeutic microRNA from the iPSC to neighboring cells in order to block the invasiveness and clinical aggravation of different forms of cancer.

## The Use of Gap Junctional Shuttling for Glioblastoma Therapy

**FIGURE 1:**
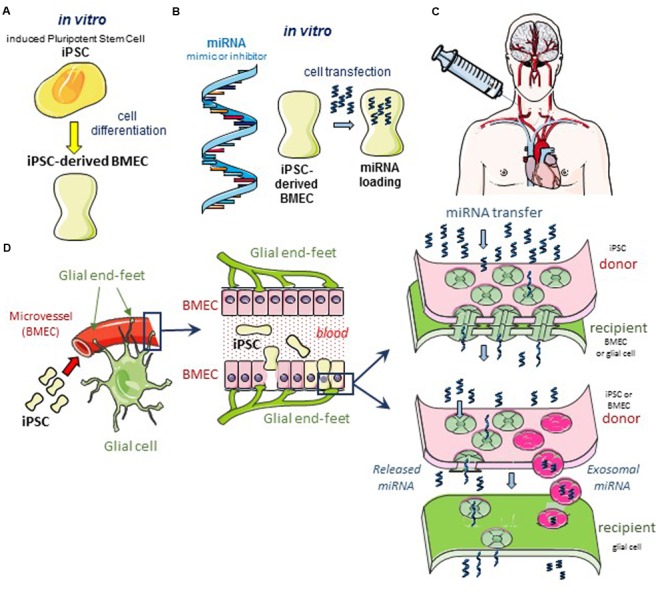
Hypothetical cell based therapeutic intervention in glioblastoma. **(A)** Induced pluripotent stem cells (iPSC) of human origin (i.e., umbilical cord, bone marrow, biopsied tissue sample from diseased patient, or the immortalized cell line hBMEC/D3) are pre-differentiated with neural cells and retinoic acid *in vitro* (Lippmann et al., 2013, 2014a). After 2 days in co-culture, cells sorting with anti-von Willebrand Factor (vWF) antibody are iPSC-derived BMEC. These cells can be sub-cultured as a pure monolayer expressing typical endothelial and BBB markers. **(B)** In the following day, iPSC-derived BMEC are loaded with the microRNA (miRNA) of interest, using the lipofectamine transfection procedure as previously described (Thuringer et al., 2016a). **(C)** After 12 h in culture, transfected cells are dissociated and suspended in a conventional infusion medium then injected directly into the patient’s carotid artery. **(D)** Schematic diagrams of the GJ-mediated shuttling of microRNA at the microvascular level *in situ*. The blue box areas are enlarged in the scheme below. Note the expected diffusion of miRNA from the iPSC-derived BMEC (yellow) to a large number of cells (bystander effect). Two modes of miRNA transfer from BMEC to glioblastoma cells are proposed: direct *via* gap-junction channels (upper panel), and indirect *via* the release of miRNA either free or contained in exosomes, to the intercellular space (lower panel). In the two modes, the Cx43 expression is required at the plasma membrane of cells, forming gap junction or hemichannel (Soares et al., 2015; De Bock et al., 2017).

Glioblastomas are the most prevalent and aggressive brain cancer and arise from glial cells. The diffuse invasive nature of glioblastoma precludes its complete surgical resection, which inevitably leads to tumor recurrence and patient death. Since the first histological observations of Hans Scherer (Scherer, 1940), the occurrence of perivascular invasion has been described in multiple experimental tumor models (Farin et al., 2006; Winkler et al., 2009). This invasion is more important in vascular endothelial growth factor (VEGF)-deficient glioblastoma cells (Blouw et al., 2003; Du et al., 2008) and brain tumor xenografts treated with anti-VEGF blocking antibodies such as bevacizumab (Rubenstein et al., 2000; Kunkel et al., 2001; de Groot et al., 2010; Carbonell et al., 2013; Baker et al., 2014). Clinical glioblastomas resistant to the anti-VEGF bevacizumab therapy also show a tendency toward increased perivascular invasion (Clark et al., 2012). Altogether, neoangiogenesis is dispensable for brain tumor progression and antiangiogenic drugs fail to meaningfully extend survival of patients with a glioblastoma. Conversely, this perivascular invasion could offer an opportunity to deliver effective drugs to cancer cells and our hypothesis is that engineered BMEC could be used for that purpose and deliver microRNA that limit cancer cell invasion and tumor growth (**Figure 1**).

### Biocompatibility

To develop such a therapeutic strategy, the first parameter to deal with is biocompatibility. A first approach consists of isolating and culturing primary BMEC collected from patient tissue samples. Adult BMEC have been cultured successfully by many laboratories but rapidly lose their phenotype (Kniesel and Wolburg, 2000; Roux and Couraud, 2005; Lyck et al., 2009). In addition, given that brain vasculature comprises only 0.1% of the brain by volume, such techniques require a significant amount of brain material to achieve a reasonable yield of BMEC, limiting high throughput applications. A scalable alternative is the use of immortalized brain endothelial cell lines such as the hCMEC/D3 human brain endothelial cell line (Weksler et al., 2005). While these cells maintain many aspects of their primary BMEC counterparts and represent very useful tools for certain applications, they lack significant barrier function (Ogunshola, 2011; Naik and Cucullo, 2012).

Induced pluripotent stem cells may be another alternative. These cells are currently explored in the treatment of a wide variety of diseases, given their ability to differentiate into every cell type (Kimbrel and Lanza, 2016). Lippmann colleagues have established the method to generate BMEC from human iPSC by co-differentiation with neural cells and retinoic acid, leading to differentiated cells which exhibit properties similar to those of tissue-derived BMEC (Lippmann et al., 2012, 2013, 2014a; Hollmann et al., 2017). Some projects aim to use iPSC to recapitulate 3D human neuron/neurovasculature interactions ‘on a chip’ *in vitro* and reconstitute the neurovascular unit, allowing pharmacological drug testing on cells derived from patients of different ages, metabolic conditions or neuro-pathologies (Brown et al., 2015; Walter et al., 2016).

Undifferentiated human embryonic stem cells express mRNA for almost all known Cx subtypes and display intercellular dye transfer, which is characteristic of GJIC (Huettner et al., 2006). iPSC obtained through reprogramming somatic fibroblasts cells also express most Cx subtypes (Oyamada et al., 2013) and stem cells of various sources express Cx43 (Kar et al., 2012). In undifferentiated cord-blood-derived iPSC, the gap junction plaques mostly contain Cx43 (Beckmann et al., 2016). Cx43 is upregulated during the reprogramming process and Cx43 knockdown via short interfering RNA significantly impairs reprogramming efficiency (Sharovskaya et al., 2012; Ke et al., 2013).

The expression of Cx43 in reprogrammed cells suggests that circulating iPSC may adhere and form Cx43 gap junction plaques with BMEC, then integrate the microvascular endothelium. We have reported that dissociated HMEC (donor), pre-loaded with a fluorescent dye, then plated onto unlabeled HMEC monolayer (acceptor), were rapidly integrated into the monolayer and formed functional gap junctions with HMEC within 2 h (Thuringer et al., 2016a). Actually, all the cells expressing Cx43 proteins, such as SW480 colon cancer cells and human monocytes, communicate with the microvascular endothelium and may pass through the monolayer (Thuringer et al., 2015a,b). Of note, glioma cells also establish Cx43 gap junction with HMEC, thereby reinforcing the barrier (Thuringer et al., 2016a).

### Route of Administration

Another question regarding the delivering of iPSC to reconstitute BMEC is the route of administration. Until now, gene vectors have been injected directly into the brain to circumvent the BBB (Do Thi et al., 2004; Yang et al., 2013); but the innate risk due to direct intra-brain administration makes this method hardly applicable in clinics. Convection-enhanced delivery, in which one or more catheters are carefully placed in the brain parenchyma for therapeutic delivery, may be a solution, this technology is currently tested in phase III clinical trial in patients with a glioblastoma (Debinski and Tatter, 2009). If validated, this approach could be used also for future iPSC-based therapies. A less complex although still tricky strategy would be to inject iPSC directly into the carotid artery, knowing that the arterial pressure is strong enough to prevent the cell adhesion in its duct.

### What Should We Transfer?

A third question is the identification of the microRNA to transfer to diseased cells through iPSC-derived BMEC (Lopez-Ramirez et al., 2016; Shea et al., 2016). The transfer of mature miR-4519 and miR-5096 from glioma cells to astrocytes enhance the glioma pro-invasive potential (Hong et al., 2015), e.g., several microRNAs associated with survival and chemotherapy resistance passed through gap junctions formed between astrocytes and lung tumor cells *in vitro* (Menachem et al., 2016). Conversely, the transfer of miR-124-3p between transfected and non-transfected glioma cells has anti-proliferative effects (Suzhi et al., 2015), and the transfer of miR-145-5p to glioma cells has anti-tumor properties (Thuringer et al., 2016a,b). Additionally, some microRNAs regulate expression and/or function of Cx for several types of cancer (Calderon and Retamal, 2016). These observations suggest that iPSC will have to be engineered to express and transfer not only selected microRNA with antitumor effects but also inhibitors of specific pro-tumorigenic microRNAs (anti-miRs) (Gurwitz, 2016).

### Efficacy

Finally, the mode of transfer between engineered iPSC and tumor cells will have to be determined as GJIC-mediated microRNA transfer may be more efficient than microvesicle/exosome-based intercellular transport (Zong et al., 2016). As indicated above, Cx43, which is highly expressed in many cell types, is involved in both GJIC-mediated and exosome-mediated transfer of microRNA. The bystander effect described with suicide gene therapeutic approaches involves both GJIC formation and exosome delivery (Yamasaki and Katoh, 1988a,b; Elshami et al., 1996; Mesnil et al., 1996; Dilber and Smith, 1997; Vrionis et al., 1997; Duflot-Dancer et al., 1998; Touraine et al., 1998; Yang et al., 1998). The GJIC-mediated bystander effect can be amplified by treatments, such as intraperitoneal injection of retinoic acid, that increase GJIC (Stahl and Sies, 1998; Touraine et al., 1998; Bertram and Vine, 2005; Kong et al., 2016). Strategies that promote the formation of GIJC between iPSC-derived BMEC and target cells in the brain may therefore increase the efficacy of this therapeutic approach.

## Conclusion

The use of iPSC is an emerging strategy that remains to be validated in the treatment of various diseases. Because the BBB is a limitation to the treatment of neurological diseases including brain cancer, iPSC-derived BMEC engineered to transfer microRNA, anti-miR or siRNA to target cells could be one of these new therapeutic applications. The transfer of selected single strand RNA through Cx43-containing gap junction channels between iPSC-derived BMEC and neuronal target cells could potentially improve the control of neurological diseases through modulation of gene expression and function in target cells. An unresolved issue is the best small RNA to transfer via GJIC, as it has to respect Cx43 expression and limit target cell development. Several practical issues have now to be solved to develop this approach to treat glioblastoma and potentially other neurological diseases.

## Author Contributions

DT: designed, drafted and revised the manuscript; ES: drafted and revised the manuscript; CG: revised the final manuscript.

## Conflict of Interest Statement

The authors declare that the research was conducted in the absence of any commercial or financial relationships that could be construed as a potential conflict of interest.
